# Effects of the Number of Neoadjuvant Cycles and Addition of Adjuvant Chemotherapy on the Prognosis of Muscle‐Invasive Bladder Cancer Treated With Radical Cystectomy

**DOI:** 10.1002/cam4.70782

**Published:** 2025-04-29

**Authors:** Shingo Hatakeyama, Rikiya Taoka, Jun Miki, Ryoichi Saito, Wataru Fukuokaya, Yoshiyuki Matsui, Takashi Kawahara, Ayumu Matsuda, Taketo Kawai, Minoru Kato, Tomokazu Sazuka, Takeshi Sano, Fumihiko Urabe, Soki Kashima, Hirohito Naito, Yoji Murakami, Makito Miyake, Kei Daizumoto, Yuto Matsushita, Takuji Hayashi, Junichi Inokuchi, Yusuke Sugino, Ken‐ichiro Shiga, Noriya Yamaguchi, Motohiro Taguchi, Keiji Yasue, Takashige Abe, Shotaro Nakanishi, Katsuyoshi Hashine, Masato Fujii, Kiyoaki Nishihara, Hiroaki Matsumoto, Shuichi Tatarano, Koichiro Wada, Sho Sekito, Ryo Maruyama, Naotaka Nishiyama, Hiroyuki Nishiyama, Hiroshi Kitamura, Chikara Ohyama, the Japanese Urological Oncology Group

**Affiliations:** ^1^ Department of Urology Hirosaki University Graduate School of Medicine Aomori Japan; ^2^ Department of Urology, Faculty of Medicine Kagawa University Kagawa Japan; ^3^ Department of Urology Jikei University Kashiwa Hospital Chiba Japan; ^4^ Department of Urology The Jikei University School of Medicine Tokyo Japan; ^5^ Department of Urology and Andrology Kansai Medical University Osaka Japan; ^6^ Department of Urology National Cancer Center Hospital Tokyo Japan; ^7^ Department of Urology, Faculty of Medicine University of Tsukuba Tsukuba Japan; ^8^ Department of Urology Graduate School of Medicine, the University of Tokyo Tokyo Japan; ^9^ Department of Urology Teikyo University School of Medicine Tokyo Japan; ^10^ Department of Urology Graduate School of Medicine, Osaka Metropolitan University Osaka Japan; ^11^ Department of Urology Graduate School of Medicine, Chiba University Chiba Japan; ^12^ Department of Urology Kyoto University Graduate School of Medicine Kyoto Japan; ^13^ Department of Urology Jikei University Hospital Tokyo Japan; ^14^ Department of Urology Akita University Graduate School of Medicine Akita Japan; ^15^ Department of Urology Kurashiki Central Hospital Okayama Japan; ^16^ Department of Urology Graduate School of Life Science, Kumamoto University Kumamoto Japan; ^17^ Department of Urology Nara Medical University Nara Japan; ^18^ Department of Urology Tokushima University Graduate School of Biomedical Sciences Tokushima Japan; ^19^ Department of Urology Hamamatsu University School of Medicine Shizuoka Japan; ^20^ Department of Urology Osaka International Cancer Institute Osaka Japan; ^21^ Department of Urology Graduate School of Medical Sciences, Kyushu University Fukuoka Japan; ^22^ Department of Nephro‐Urologic Surgery and Andrology Mie University Graduate School of Medicine Mie Japan; ^23^ Department of Urology Harasanshin General Hospital Fukuoka Japan; ^24^ Department of Urology Tottori University Faculty of Medicine Tottori Japan; ^25^ Department of Urology Hyogo Medical University Hyogo Japan; ^26^ Department of Urology Jikei University Katsushika Medical Center Tokyo Japan; ^27^ Department of Renal and Genitourinary Surgery, Graduate School of Medicine Hokkaido University Sapporo Hokkaido Japan; ^28^ Department of Urology, Graduate School of Medicine University of the Ryukyus Okinawa Japan; ^29^ Department of Urology NHO Shikoku Cancer Center Matsuyama Ehime Japan; ^30^ Department of Urology Faculty of Medicine, University of Miyazaki Miyazaki Japan; ^31^ Department of Urology Kurume University School of Medicine Fukuoka Japan; ^32^ Department of Urology Graduate School of Medicine Yamaguchi University Yamaguchi Japan; ^33^ Department of Urology, Graduate School of Medical and Dental Sciences Kagoshima University Kagoshima Japan; ^34^ Department of Urology Shimane University Faculty of Medicine Matsue Japan; ^35^ Department of Urology Aichi Cancer Center Hospital Nagoya Japan; ^36^ Department of Urology Niigata University Graduate School of Medicine Niigata Japan; ^37^ Department of Urology, Faculty of Medicine University of Toyama Toyama Japan

**Keywords:** adjuvant chemotherapy, bladder cancer, neoadjuvant chemotherapy, overall survival, radical cystectomy

## Abstract

**Objective:**

To evaluate the effects of the number of neoadjuvant chemotherapy (NAC) cycles and the addition of adjuvant chemotherapy (AC) after NAC on overall survival (OS) of patients with muscle‐invasive bladder cancer (MIBC).

**Patients and Methods:**

We retrospectively evaluated 1687 patients with cT2‐4NxM0 MIBC who received radical cystectomy (RC) alone or RC plus perioperative chemotherapy at 36 institutions within the Japanese Urological Oncology Group. We evaluated the effect of the number of NAC cycles (2 vs. ≥ 3 cycles) and the addition of AC on OS.

**Results:**

Among the 1687 patients analyzed, 946 received a median of three NAC cycles. The pathologic complete response rate did not significantly differ between those who received 2 (22.9%) and ≥ 3 cycles (27.5%, *p* = 0.112). Moreover, no significant difference in OS was observed between the groups (*p* = 0.559). Multivariable Cox regression analysis showed that pathologic high‐risk (ypT2–4, pT3–4, or pN+) or cisplatin ineligibility were significantly associated with poor OS but not the number of NAC cycles (*p* = 0.238). We identified 942 pathologically high‐risk patients after RC who were eligible for AC. Notably, no significant OS improvement was observed with the addition of AC as intensive perioperative chemotherapy after NAC. The primary limitation was selection bias from confounding by clinical indication.

**Conclusions:**

Our findings showed that three or more NAC cycles and the addition of AC may have limited effects on OS in MIBC patients who received RC.

## Abstract 2

We found the effect of the number of neoadjuvant chemotherapy cycles (2 vs. ≥ 3 cycles) achieve similar benefits, and the addition of adjuvant chemotherapy after neoadjuvant chemotherapy may have a limited effect on OS in muscle‐invasive bladder cancer.

## 1 Introduction

Perioperative chemotherapy and radical cystectomy (RC) have been the standards of care for patients with muscle‐invasive bladder cancer (MIBC) [1, 2, 3, 4, 5]. However, survival outcomes after RC remain poor, with patients having a 5‐year overall survival (OS) of only 50%–60% [6, 7]. Neoadjuvant chemotherapy (NAC) and adjuvant chemotherapy (AC) are among the available strategies to improve outcomes [8, 9], but the optimal number of cycles and the benefit of additional AC after NAC have not been well elucidated [10, 11]. Furthermore, the efficacy of postoperative adjuvant immunotherapy, as reported in the CheckMate‐274 trial, has reaffirmed the importance of postoperative adjuvant immunotherapy after NAC [12]. The current study therefore aimed to evaluate the effects of perioperative therapy, particularly the number of NAC cycles and the addition of AC, on the prognosis of patients with MIBC.

## 2 Methods

This multi‐institutional study was approved by the institutional review boards of all 36 participating institutions within the Japanese Urological Oncology Group. Data were collected from 2674 patients with BC who underwent RC between January 2013 and December 2019 at the participating institutions. The cutoff date for data collection was December 31, 2021. This study was approved by the ethics committee of Kagawa University School of Medicine (2021‐140) and of all participating hospitals. Written consent was not obtained in exchange for public disclosure of study information (opt‐out approach).

### 2.1 Selection of Patients

**FIGURE 1:**
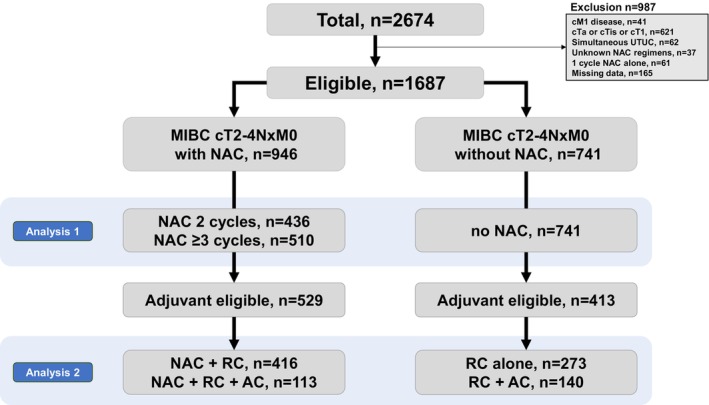
Flowchart for patient selection.

We excluded 987 patients with metastatic disease (*n* = 41), cTa‐1 (*n* = 621), concurrent upper‐tract urinary cancer (*n* = 62), unknown NAC regimens (*n* = 37), a single cycle of NAC alone (*n* = 61), and data missing (*n* = 165). Finally, 1687 eligible patients with cT2‐4NxM0 disease who received RC alone or perioperative chemotherapy + RC were included. We divided the patients into three groups: those who received two cycles of NAC (*n* = 436), those who received three or more cycles of NAC (*n* = 510), and those who did not receive NAC (*n* = 741). Furthermore, we selected 942 patients with pathologic high‐risk factors (either ypT2–4, pT3–4, or pN+) who were eligible for adjuvant therapy (Figure 1).

### 2.2 Data Collection

Clinical, pathologic, and survival outcomes were retrospectively collected from the medical records at each of the 36 participating institutions. The following variables were collected and analyzed: age, sex, Eastern Cooperative Oncology Group performance status (ECOG PS), estimated glomerular filtration rate (eGFR) [13], number of NAC cycles, types of NAC regimens, clinical stage, pathologic stage, and OS. Tumor stage and grade were stratified according to the eighth edition of the TNM classification. OS was defined from the time of RC to death/final follow‐up in patients without NAC and from the time of NAC to death/final follow‐up in patients who received NAC.

### 2.3 Surgical Procedures

RC and lymph node dissection were performed using a basic technique. Given the multicenter design of the current study, the indications for surgery, surgical techniques, and extent of lymph node dissection were not standardized. The surgical modality, urinary diversion, and extent of dissection were determined by the attending physician.

### 2.4 Platinum‐Based Neoadjuvant or Adjuvant Chemotherapy

Patients received either gemcitabine plus cisplatin (GCis); gemcitabine plus carboplatin (GCarbo), methotrexate, vinblastine, doxorubicin, and cisplatin (MVAC); or dose‐dense MVAC (ddMVAC) [14, 15]. Regimens were selected based on our guideline for cisplatin eligibility [16]. The eligibility criteria for cisplatin in Japan are urothelial carcinoma including tumor stage cT2–4aNanyM0, eGFR ≥ 50–60 mL/min (depending on primary urologists), and ECOG PS 0–1. Indications for NAC were MIBC ≥ cT2 or cN+ disease. Cycles were repeated every 14–28 days in the NAC setting. AC was indicated when pathologic high‐risk factors (ypT2–4, pT3–4, or pN+) were observed. We administered AC in selected patients with feasible postoperative status for toxic chemotherapy.

### 2.5 Outcome Analysis

We investigated the proportion of NAC cycles in our real‐world practice. The effects of the number of NAC cycles on pathologic response rate and OS were determined by comparing those who received 2 and ≥ 3 NAC cycles. Furthermore, the effect of delayed surgery (days from NAC to RC) on OS was determined by comparing those who underwent surgery in ≤ 90 and > 90 days.

The AC administration rate for eligible patients (ypT2–4, pT3–4, or pN+) was determined by comparing patients who received RC + AC and NAC + RC + AC. The effects of AC in addition to NAC on OS were determined by comparing those who received RC + no AC and RC + AC, as well as those who received NAC + no AC and NAC + AC.

### 2.6 Statistical Analyses

Statistical analyses were performed using BellCurve for Excel 3.10 (Social Survey Research Information Co. Ltd., Tokyo, Japan), GraphPad Prism 7.00 (GraphPad Software, San Diego, CA, USA), and R 4.0.2, a Language and Environment for Statistical Computing (The R Foundation, Vienna, Austria). Intergroup differences were determined using Student's *t* test or Mann–Whitney *U* test. Fisher's exact test or chi‐square (*χ* ^2^) test was used to compare categorical variables. Quantitative variables were expressed as means with standard deviations or medians with interquartile ranges. The OS rate from initial treatment until death/final follow‐up was estimated using the log‐rank test. Multivariable Cox regression proportional hazard models were used to determine the effects of the number of neoadjuvant cycles and the addition of AC on OS. We have labeled the factors that tend to have a poor prognosis as follows: adjuvant eligible = 1 and cisplatin‐ineligible = 1. Cisplatin eligibility criteria are defined for patients with metastatic bladder cancer by the Galsky criteria [16], including renal function, poor ECOG PS, severe neuropathy, severe hearing loss, or severe heart failure. The cisplatin ineligibility classification was adopted as an independent factor, regardless of whether cisplatin was administered or not. Hazard ratios with 95% confidence intervals (CIs) were calculated after controlling for potential confounders.

## 3 Results

A total of 1687 patients with cT2‐4NxM0 who received RC alone (*n* = 741) or perioperative chemotherapy + RC (*n* = 946) were identified. The median age and follow‐up were 70.8 years and 37.6 months, respectively. The background characteristics of patients are summarized in Tables 1 (all patients) and 2 (those who received NAC). Notably, significant differences in age, ECOG PS, eGFR, cisplatin eligibility, variant histology, cT stage, cN stage, laparoscopic surgery, and adjuvant eligibility were observed between the patients with and without NAC.

**TABLE 1:** Background of patients.

	RC without NAC	NAC + RC	*p*
*n*	741	946	
Median age, years (IQR)	73 (67–79)	70 (64–75)	< 0.001
Sex (Male), *n*	576 (78%)	747 (79%)	0.542
ECOG PS > 0, *n*	235 (32%)	204 (22%)	< 0.001
eGFR, mL/min/1.73 m^2^ (IQR)	59.3 (43.6–73.8)	64.7 (53.0–78.8)	< 0.001
Cisplatin ineligible, *n*	271 (37%)	186 (20%)	< 0.001
NAC cycles (IQR)	0	3 (2–3)	
Cisplatin‐based regimens, *n*	0	798 (84%)	
Clinical stage, *n*			
cT2	513 (69%)	465 (49%)	< 0.001
cT3 or 4	228 (31%)	481 (51%)	
cN+	47 (6.3%)	173 (18%)	< 0.001
Surgical procedures, *n*			
Open	521 (70%)	565 (60%)	< 0.001
Laparoscopic surgery	146 (20%)	206 (22%)	
Robotic surgery, *n*	72 (9.7%)	175 (19%)	
Urinary diversion (Neobladder)	102 (14%)	201 (21%)	< 0.001
Pathologic outcomes, *n*			
Tumor grade 2–3 or high‐grade	675 (91%)	857 (91%)	0.724
Variant histology, *n*	174 (23%)	165 (17%)	0.002
pT0N0	83 (11%)	222 (24%)	< 0.001
pN+	176 (24%)	178 (19%)	< 0.001
AC eligible, *n*	150 (20%)	529 (56%)	< 0.001
Deceased, *n*	240 (32%)	252 (27%)	
Median follow‐up, months (IQR)	36 (13–66)	39 (21–61)	

**TABLE 2:** Background of patients with NAC.

	NAC 2 cycles	NAC ≥ 3 cycles	
*n*	436	510	
Median age, years (IQR)	70 (65–75)	68 (64–74)	0.003
Sex (Male), *n*	341 (78%)	406 (80%)	0.599
ECOG PS > 0, *n*	44 (10%)	54 (11%)	0.803
eGFR, mL/min/1.73 m^2^ (IQR)	65.8 (52.9–78.0)	63.8 (53.2–79.4)	0.719
Cisplatin ineligible, *n*	87 (20%)	99 (19%)	0.834
NAC cycles (IQR)	2 (2–2)	3 (3–3)	
Cisplatin‐based regimens, *n*	340 (78%)	458 (90%)	< 0.001
Clinical stage, *n*			
cT2	226 (52%)	239 (47%)	0.127
cT3 or 4	201 (48%)	271 (53%)	0.064
cN+	49 (11%)	124 (24%)	< 0.001
Surgical procedures, *n*			
Open	294 (67%)	281 (55%)	< 0.001
Laparoscopic surgery	79 (18%)	127 (25%)	
Robotic surgery, *n*	73 (17%)	102 (20%)	
Urinary diversion (Neobladder)	111 (26%)	90 (18%)	0.003
Pathologic outcomes, *n*			
Tumor grade 2–3 or high‐grade	391 (90%)	466 (91%)	0.374
Variant histology, *n*	70 (16%)	95 (19%)	0.299
pT0N0	94 (22%)	128 (25%)	0.201
pN+	85 (19%)	93 (18%)	0.621
Adjuvant eligible, *n*	238 (55%)	291 (57%)	0.445
Deceased, *n*	121 (28%)	131 (26%)	
Median follow‐up, months (IQR)	40 (20–60)	39 (22–61)	

**FIGURE 2:**
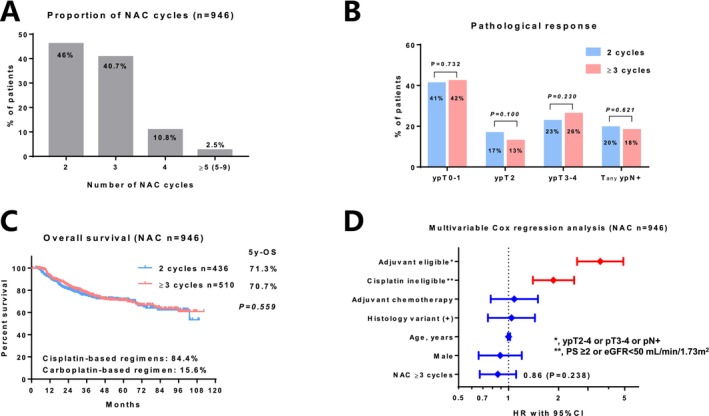
The effects of the number of neoadjuvant chemotherapy (NAC) cycles on outcomes. (A) Proportion of NAC cycles in 946 patients who received NAC. (B) Pathologic response in patients who received 2 and ≥ 3 cycles of NAC. (C) Comparison of the overall survival between patients who received 2 and ≥ 3 cycles of NAC. (D) Multivariable Cox regression analysis for overall survival.

Among the 946 patients who received NAC, 46%, 40.7%, 10.8%, and 2.5% received 2, 3, 4, and ≥ 5 cycles, respectively (Figure 2A). In this cohort, the median number of NAC cycles was 3. The number of patients who received cisplatin‐based and carboplatin‐based regimens was 796 (84.4%) and 148 (15.6%), respectively. The pathologic complete response rate (ypT0) did not significantly differ between patients who received 2 (*n* = 100/436, 22.9%) and ≥ 3 cycles (*n* = 140/510, 27.5%; *p* = 0.112) of NAC. Similarly, the rate of patients with ypT0–1, ypT2, ypT3–4, and T_any_pN+ did not significantly differ between those who received 2 and ≥ 3 cycles of NAC (Figure 2B). The unadjusted OS did not significantly differ between patients who received 2 and ≥ 3 cycles of NAC (5‐year OS, 71.3% vs. 70.7%; *p* = 0.559, Figure 2C). Multivariable Cox regression analysis showed that pathologic high‐risk disease (ypT2–4, pT3–4, or pN+) or cisplatin ineligibility (poor performance status, impaired renal function, or comorbidities contraindicating chemotherapy) was significantly associated with poor OS, but not the number of NAC ≥ 3 cycles (hazard ratio, 0.86; 95% CI, 0.67–1.11; *p* = 0.238; Figure 2D).

The 5‐year OS was 71.8%, 66.5%, and 72.7% in those who received 3, 4, and ≥ 5 cycles of NAC, respectively (Figure S1A). No significant difference in OS was observed between patients who received 2 and ≥ 3 cycles of NAC, regardless of whether they received cisplatin‐based (Figure S1B) or carboplatin‐based regimens (Figure S1C).

The median time from NAC initiation to RC was 3.0 months (Figure S2A). No significant difference in OS was observed between patients who underwent RC after ≤ 90 and > 90 days (Figure S2B). The outcomes stratified by NAC regimens are shown in Figure S3. We identified patients with GCis, MVAC/ddMVAC, and GCarbo as 758, 40, and 148, respectively. The median age (Figure S3A) and eGFR (Figure S3B) were significantly older and lower in the patients with GCarbo than those with GCis/MVAC/ddMVAC. Based on the unfavorable background differences, OS was considerably shorter in the patients with GCarbo than in those with GCis/MVAC/ddMVAC. The 5‐year OS in patients with GCis, MVAC/ddMVAC, and GCarbo was 74.0%, 67.4%, and 57.1%, respectively (Figure S3C).

**FIGURE 3:**
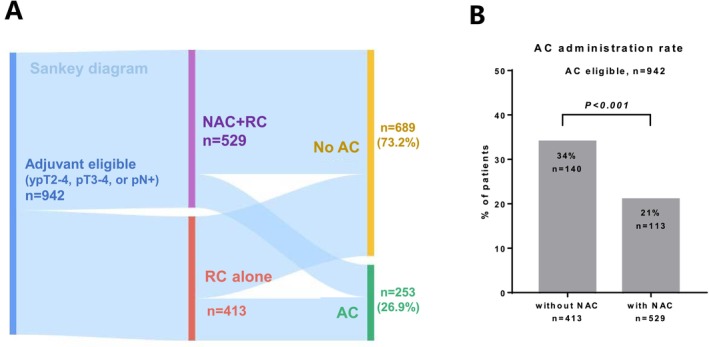
Adjuvant chemotherapy (AC) administration rate for patients eligible for AC. (A) Sankey diagram for treatment flow in 942 patients with pathologic high‐risk features (ypT2‐4, pT3‐4, or pN+). (B) AC administration rate in patients with and without NAC.

**FIGURE 4:**
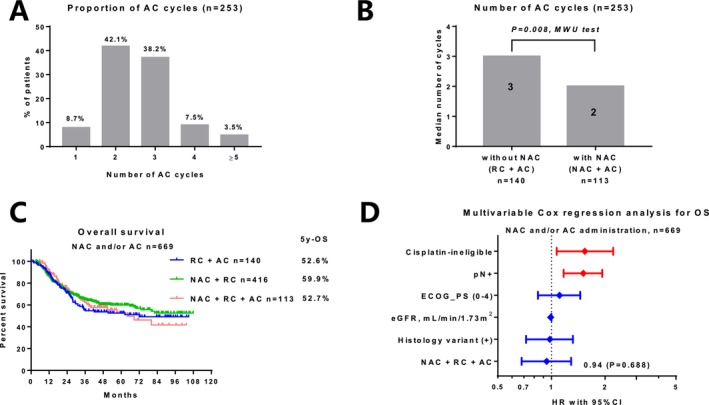
Effects of adding adjuvant chemotherapy (AC) after neoadjuvant chemotherapy (NAC) in patients eligible for AC. (A) Proportion of AC cycles in 253 patients who received AC. (B) The number of AC cycles between patients with and without NAC. (C) Overall survival among the 669 patients who received NAC and/or AC. (D) Multivariable Cox regression analysis for overall survival among the 669 patients who received NAC and/or AC.

We identified 942 pathologically high‐risk patients after RC who were eligible for AC. The background characteristics of the identified patients are summarized in Table 3. We presented the treatment flow using a Sankey diagram (Figure 3A). AC was administered in 253 (26.9%) patients. The usage rates of cisplatin‐based regimens for NAC and AC were 83.2% and 79.9%, respectively. The administration rate of AC was significantly higher in patients without NAC (34%) than in those who received NAC (21%) (*p* < 0.001; Figure 3B). Among the 253 patients who received AC, 8.7%, 42.1%, 38.2%, 7.5%, and 3.5% received 1, 2, 3, 4, and ≥ 5 AC cycles, respectively (Figure 4A). The number of AC cycles differed significantly between patients with (median 2) or without (median 3) NAC (*p* = 0.008; Figure 4B). The 5‐year OS in patients with RC + AC, NAC + RC, and NAC + RC + AC was 52.6%, 59.9%, and 52.7%, respectively (Figure 4C). The addition of AC was not significantly associated with OS improvement in patients with (*p* = 0.556, Figure S4A) or without (*p* = 0.122, Figure S4B) NAC. Multivariable Cox regression analysis showed that the addition of AC after NAC was not significantly associated with OS improvement among the patients who received NAC and/or AC (*n* = 669) (hazard ratio, 0.94; 95% CI, 0.68–1.29; *p* = 0.688; Figure 4D).

**TABLE 3:** Background of AC eligible patients.

	RC alone	RC + AC	NAC + RC	NAC + RC + AC
*n*	273	140	416	113
Median age, years (IQR)	76 (69–81)	71 (66–76)	70 (65–75)	68 (63–7)
Sex (Male), *n*	196 (72%)	106 (76%)	318 (76%)	87 (77%)
ECOG PS > 0, *n*	112 (41%)	39 (28%)	57 (14%)	13 (12%)
eGFR, mL/min/1.73m^2^ (IQR)	52.2 (35.8–66.6)	59.4 (48.3–73.6)	62.6 (51.5–79.8)	61.6 (49.0–78.7)
Cisplatin ineligible, *n*	141 (52%)	40 (29%)	97 (23%)	31 (27%)
NAC cycles (IQR)			3 (2–2)	3 (2–3)
Cisplatin‐based regimens, *n*			348 (84%)	92 (81%)
Clinical stage, *n*				
cT2	164 (60%)	79 (56%)	169 (41%)	41 (36%)
cT3 or 4	110 (40%)	61 (44%)	247 (59%)	72 (64%)
cN+	22 (8.1%)	19 (14%)	90 (22%)	27 (24%)
Surgical procedures, *n*				
Open	196 (72%)	112 (80%)	270 (65%)	67 (59%)
Laparoscopic surgery	58 (21%)	21 (15%)	77 (19%)	22 (19%)
Robotic surgery, *n*	19 (7.0%)	7 (5.0%)	69 (17%)	24 (21%)
Urinary diversion (Neobladder)	20 (7.3%)	18 (13%)	70 (17%)	10 (8.8%)
Pathologic outcomes, *n*				
Tumor grade 2–3 or high‐grade	245 (90%)	124 (89%)	369 (89%)	106 (94%)
Variant histology, *n*	65 (24%)	40 (29%)	76 (18%)	25 (22%)
pN+	97 (36%)	79 (56%)	112 (27%)	66 (58%)
AC cycles, *n*		3 (2–3)		2 (2–2)
Cisplatin‐based regimens, *n*		98 (70%)		63 (56%)
Deceased, *n*	117 (43%)	64 (46%)	152 (37%)	47 (42%)
Median follow‐up, months (IQR)	17 (8–46)	35 (18–62)	33 (17–51)	35 (18–50)

We additionally analyzed the OS difference in patients with ypN− or ypN+ between the NAC + RC and NAC + RC + AC who were eligible for AC. OS was not significantly different between the NAC + RC and NAC + RC + AC in patients with ypN− (Figure S5A). However, we observed no significant but meaningful improvement of OS in patients with ypN+ in the NAC + RC + AC compared to those with NAC + RC (*p* = 0.075; Figure S5B).

## 4 Discussion

The standards of care for patients with nonmetastatic MIBC include RC and cisplatin‐based NAC in the absence of contraindications. Generally, 3–4 cycles of NAC seem to be widely accepted; however, the appropriate number of NAC cycles remains unclear. While recent studies have compared three versus four courses of NAC, they have reported controversial findings [10, 11, 17]. Accordingly, the current study was initiated to address the following four clinical questions: (1) what is the median number of NAC and AC cycles in Japanese practice, (2) what is the optimal number of courses of NAC, (3) what is the percentage of patients who receive AC after NAC, and (4) does performing AC after NAC carry any merit? Although some studies have attempted to address these questions [10, 11, 17, 18, 19], few large‐scale database studies have covered all of Japan.

This multicenter retrospective study, including 1687 cases between 2013 and 2019, showed that the median number of NAC and AC cycles in Japanese practice was 3 (IQR, 2–3) and 2 (IQR, 2–3), respectively. Our findings showed that 2 or ≥ 3 cycles of NAC had similar effects on pathologic responses and OS and that AC administration after NAC had limited benefits on OS. Delays in RC due to NAC (> 90 days) were also not significantly associated with poor OS. Multivariate Cox regression analysis suggested that pathologic unfavorable factors and/or baseline cisplatin ineligibility were significantly associated with poor OS but not the number of NAC cycles. In this cohort, 26.9% of the patients with pathologic high‐risk characteristics (AC eligible, *n* = 942) received AC. Meanwhile, 21.4% of the patients received AC after NAC (*n* = 113/529). This explains why the AC administration rate (34% vs. 21%) and the number of AC cycles (3 vs. 2 cycles) were significantly higher in patients with no NAC than in those who received NAC. Finally, we found that the addition of AC after NAC may have limited benefits. However, we also found a significant selection bias in pN+ for AC administration between those who received NAC (27%) and NAC + AC (58%; *p* < 0.001). Hence, caution should be exercised in interpreting our results given the apparent patient selection bias, especially considering the retrospective nature of the current study. There is a high possibility that the number of NAC cycles will increase in more advanced cases, and selection bias in real‐world clinical practice cannot be ruled out.

The optimal number of NAC cycles has continued to be a topic of debate. Controversial reports have been published regarding the effects of the number of NAC cycles on outcomes. Accordingly, Patel et al. [17] reported that 3 (*n* = 114) and 4 cycles of cisplatin‐based NAC (*n* = 157) demonstrated similar pathologic response and short‐term survival. In contrast, a large‐scale multicenter retrospective study (*n* = 828) suggested that 4 cycles of cisplatin‐based NAC (*n* = 444) had potential benefit on pathologic response and overall survival over 3 cycles of NAC (*n* = 384) [10]. Aydin et al. [11] also reported similar trends after comparing patients who received 4 (*n* = 59) and 3 cycles of NAC (*n* = 107). Although prior studies have compared 3 versus 4 cycles, the current study divided our cohort into two groups: those who received 2 and ≥ 3 cycles, after considering that many of our patients received 2–3 cycles of NAC. Despite the small number of NAC cycles encountered in our practice, the pathologic response rate (ypT0 rate: 22.9%–27.5%; ypT0–1 rate: 41.1%–42.2%) was comparable to those reported in other studies. Therefore, 2 cycles of NAC may be sufficient for Japanese patients with slightly lower renal function considering their small body size. Given that the VESPER study [20] demonstrated the efficacy of dose‐dense MVAC, 4–6 cycles of dose‐dense MVAC may become popular in Japanese clinical practice. The optimal cycles should be investigated in future studies considering that Japanese patients may find it difficult to complete 6 cycles given the high incidence of adverse events.

Our results showed no significant difference in OS between patients who did and did not receive AC after NAC. This finding is similar to those published in a previous study [19]. However, it is not yet clear because competing results have been reported that AC after NAC and RC can improve the prognosis [21]. Given that pathologic high‐risk disease after NAC (ypT2–4 or pN+) suggests the existence of chemotherapy‐resistant clones, the limited efficacy of AC with a similar regimen may be reasonable. A subanalysis of the CheckMate‐274 trial [12], which showed the efficacy of adjuvant immunotherapy, suggested the efficacy of immunotherapy after NAC. Although we still await the OS results for the CheckMate‐274 trial, immunotherapy after NAC could be efficacious, particularly due to immunologic cell death. Our results indirectly support the utility of adjuvant immunotherapy after NAC as the standard of care in patients with MIBC.

The difference from a similar study evaluating the impact of NAC cycles on outcomes needs to be debated. A recent study found that early cessation of planned NAC (< 3 cycles) was associated with a worse complete pathologic response rate, progression‐free survival, and OS [22]. However, as the 5‐year OS of 13.3% was too poor even in real‐world practice compared to 53.3% with ≥ 3 cycles of NAC in their study, there might be strong selection biases. As is known from many urological cancer studies, Japanese cohorts are known to have a better prognosis despite being older than those in randomized controlled trials. This is probably influenced by racial differences, differences in health insurance systems, and the differences between the departments in charge of treatment. The Japanese universal health insurance system can provide equal opportunities for treatment with a low financial burden. In addition, urologists prescribe NAC and decide the RC at the appropriate time in Japan. However, in Western countries, cooperation between oncologists and urologists is necessary. This may lead to delays in seamless treatment strategies. These advantages may be integrated, and the prognosis may be favorable in Japan.

The impact of AC for patients with ypN+ might be beneficial compared with without AC. A recent study from the Turkish Urooncology Bladder Cancer study group suggested that ypN+ showed a significantly poor prognosis in nonresponders to NAC, and ypN+ may be an important prognostic indicator for AC candidates [23]. However, with the introduction of adjuvant immunotherapy, it is unclear whether AC or immunotherapy after NAC is better for these patients with ypN+. Further investigation is needed to optimize treatment.

### 4.1 Limitations

The limitations of the current study include its retrospective design and uncontrollable selection biases for NAC and/or AC administration. Caution should be exercised in interpreting our results, given that the many differences in practice patterns between institutions may have an impact on results. We could not address chemotherapy‐related adverse events. The median follow‐up was also relatively short at 39 months. We could not address the utility and efficacy of carboplatin‐based regimens for cisplatin‐ineligible patients due to the limited number of samples and data. Despite the limitations, the current study provides additional information regarding the effects of the number of NAC cycles and the addition of AC on patient response and survival.

## 5 Conclusion

The present study suggests that patients who received 2 or ≥ 3 cycles of NAC achieved similar benefits in pathologic response and overall survival. The addition of AC after NAC may have a limited effect on OS in MIBC patients who received RC.

## Author Contributions

**Shingo Hatakeyama:** conceptualization (equal), data curation (equal), formal analysis (equal), funding acquisition (equal), investigation (equal), methodology (equal), project administration (equal), resources (equal), writing – original draft (equal), writing – review and editing (equal). **Rikiya Taoka:** conceptualization (equal), data curation (equal), writing – review and editing (equal). **Jun Miki:** data curation (equal). **Ryoichi Saito:** data curation (equal), writing – review and editing (equal). **Wataru Fukuokaya:** data curation (equal). **Yoshiyuki Matsui:** data curation (equal). **Takashi Kawahara:** data curation (equal). **Ayumu Matsuda:** data curation (equal). **Taketo Kawai:** data curation (equal). **Minoru Kato:** data curation (equal). **Tomokazu Sazuka:** data curation (equal). **Takeshi Sano:** data curation (equal). **Fumihiko Urabe:** data curation (equal). **Soki Kashima:** data curation (equal). **Hirohito Naito:** data curation (equal). **Yoji Murakami:** data curation (equal). **Makito Miyake:** data curation (equal). **Kei Daizumoto:** data curation (equal). **Yuto Matsushita:** data curation (equal). **Takuji Hayashi:** data curation (equal). **Junichi Inokuchi:** data curation (equal). **Yusuke Sugino:** data curation (equal). **Ken‐ichiro Shiga:** data curation (equal). **Noriya Yamaguchi:** data curation (equal). **Motohiro Taguchi:** data curation (equal). **Keiji Yasue:** data curation (equal). **Takashige Abe:** data curation (equal). **Shotaro Nakanishi:** data curation (equal). **Katsuyoshi Hashine:** data curation (equal). **Masato Fujii:** data curation (equal). **Kiyoaki Nishihara:** data curation (equal). **Hiroaki Matsumoto:** data curation (equal). **Shuichi Tatarano:** data curation (equal). **Koichiro Wada:** data curation (equal). **Sho Sekito:** data curation (equal). **Ryo Maruyama:** data curation (equal). **Naotaka Nishiyama:** data curation (equal). **Hiroyuki Nishiyama:** data curation (equal). **Hiroshi Kitamura:** data curation (equal). **Chikara Ohyama:** data curation (equal).

## Conflicts of Interest

The authors declare no conflicts of interest.

## Supporting information


Figures S1–S5.
**Figure S1.** Overall survival (OS) analysis according to the number of neoadjuvant chemotherapy (NAC) cycles and regimens. (A) Overall survival (OS) stratified according to the number of NAC cycles: 2, 3, 4, and ≥ 5. (B) Comparison of the OS between the patients who received 2 and ≥ 3 cycles of cisplatin‐based NAC. (C) Comparison of the OS between the patients who received 2 and ≥ 3 cycles of carboplatin‐based NAC.
**Figure S2.** Impact of delays in radical cystectomy (RC) on the OS of 939 patients who received neoadjuvant chemotherapy (NAC). (A) The median months from NAC to RC. We excluded seven cases without any data for time from NAC to RC. (B) Comparison of the overall survival between the patients with RC delays of ≤ 90 and > 90 days.
**Figure S3.** Background difference of NAC regimens and its impact on OS. (A) Age difference between the GCis, MVAC/ddMVAC, and GCarbo. (B) eGFR difference between the GCis, MVAC/ddMVAC, and GCarbo. (C) OS difference between the GCis, MVAC/ddMVAC, and GCarbo.
**Figure S4.** Subgroup analysis of the effect of adding adjuvant chemotherapy (AC) in patients eligible for AC. (A) Comparison of the OS between the patients who did and did not receive AC after NAC + RC. (B) Comparison of the overall survival between the patients who did and did not receive AC after RC.
**Figure S5.** Comparison of OS in patients with ypN− or ypN+ between the NAC + RC and NAC + RC + AC who were eligible for AC. (A) Comparison of OS in patients with ypN− between the NAC + RC and NAC + RC + AC. (B) Comparison of OS in patients with ypN+ between the NAC + RC and NAC + RC + AC.

## Data Availability

Data are not available to other researchers. Data sharing is available upon request.
