# Supplementary figures and images for: Effects of the Number of Neoadjuvant Cycles and Addition of Adjuvant Chemotherapy on the Prognosis of Muscle‐Invasive Bladder Cancer Treated With Radical Cystectomy
Source: Cancer Med. 2025 Apr 29;14(9):e70782. doi: 10.1002/cam4.70782 (PMC12041134; doi:10.1002/cam4.70782)

Fig.S1

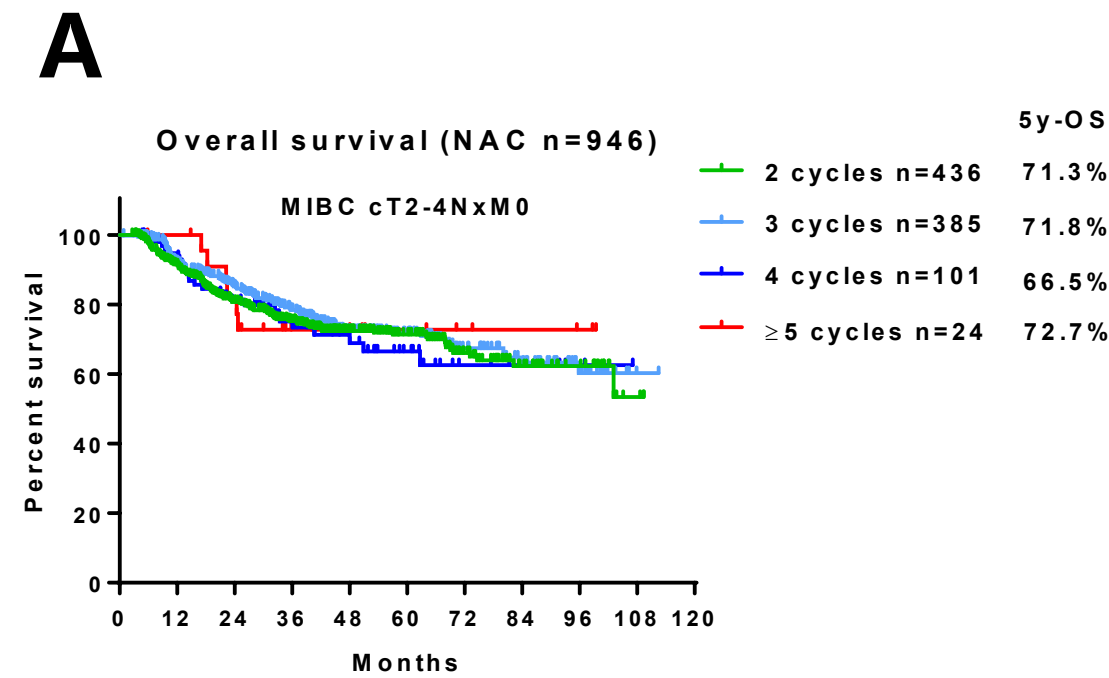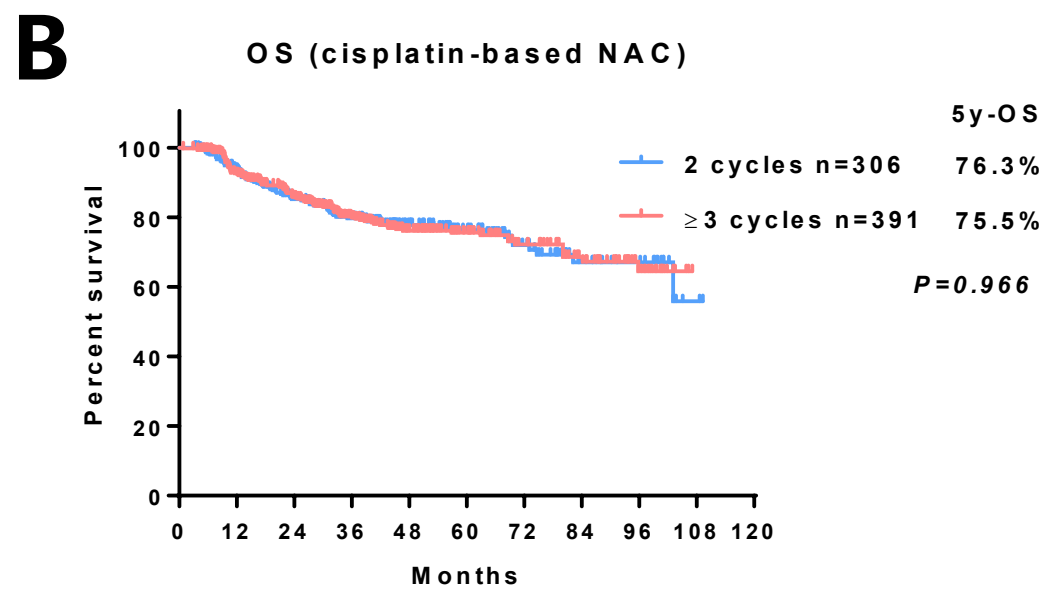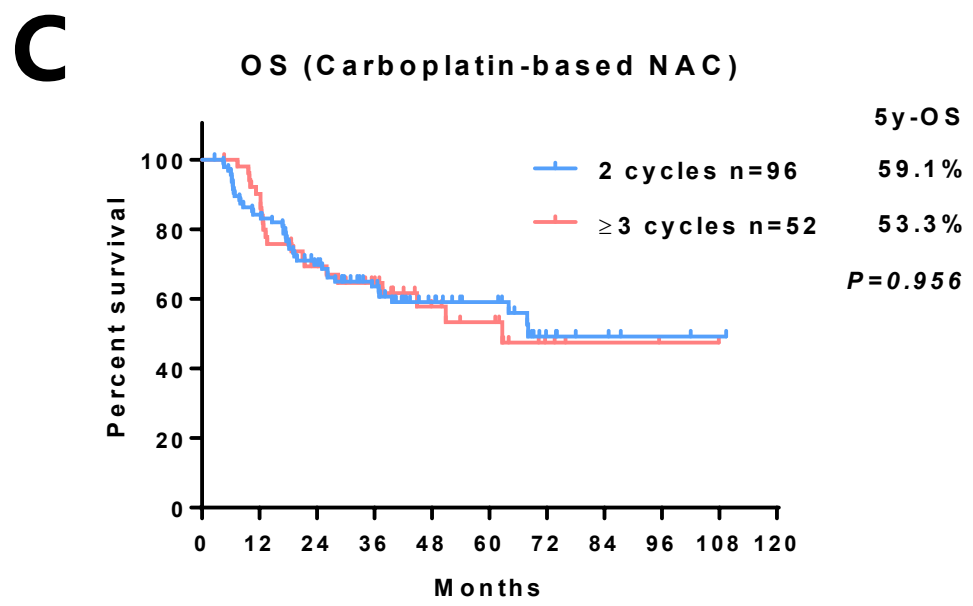

Fig.S2

A

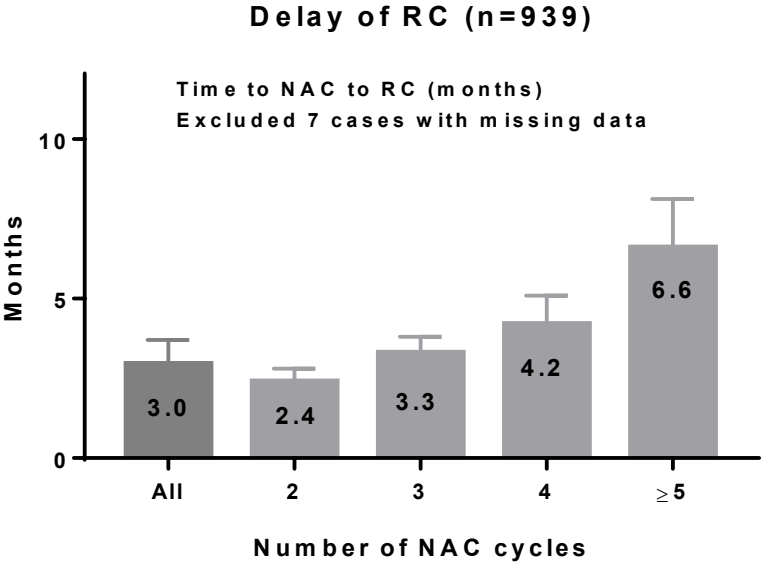

B

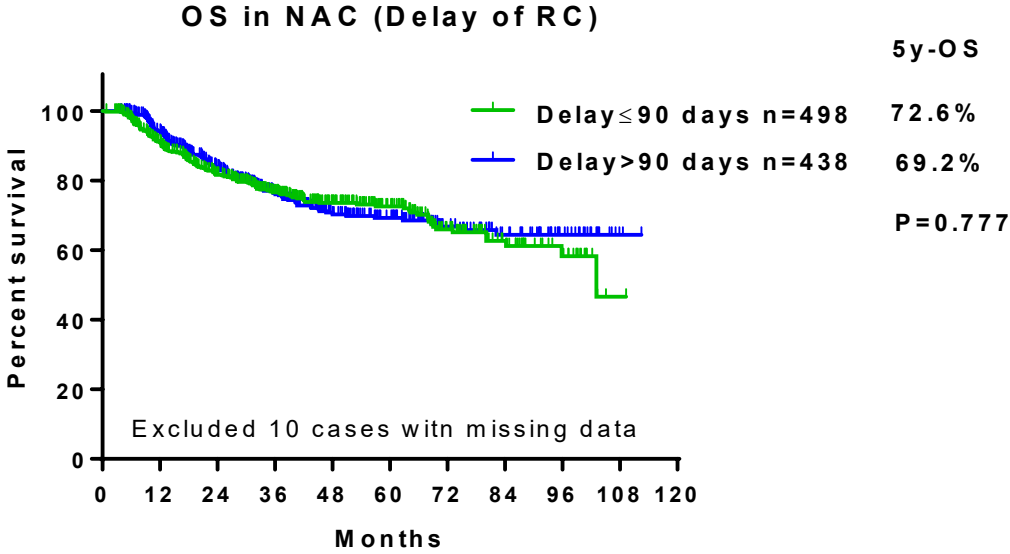

Fig.S3

A

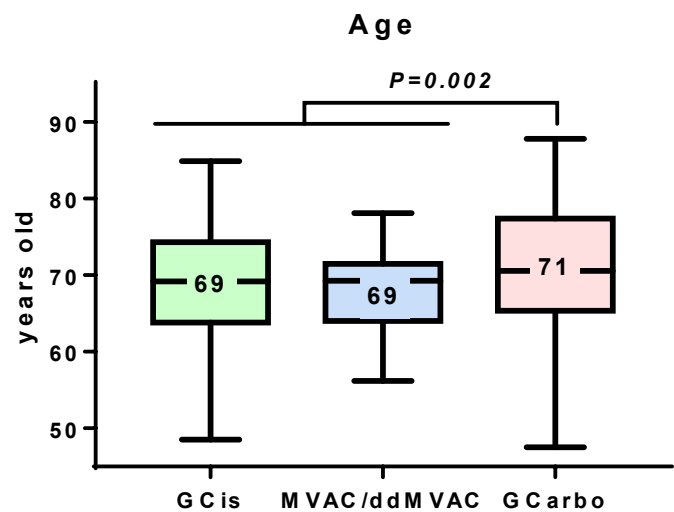

B

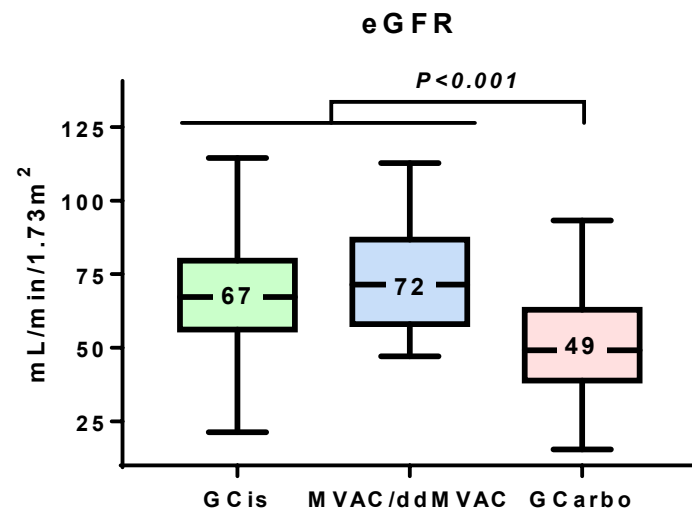

C

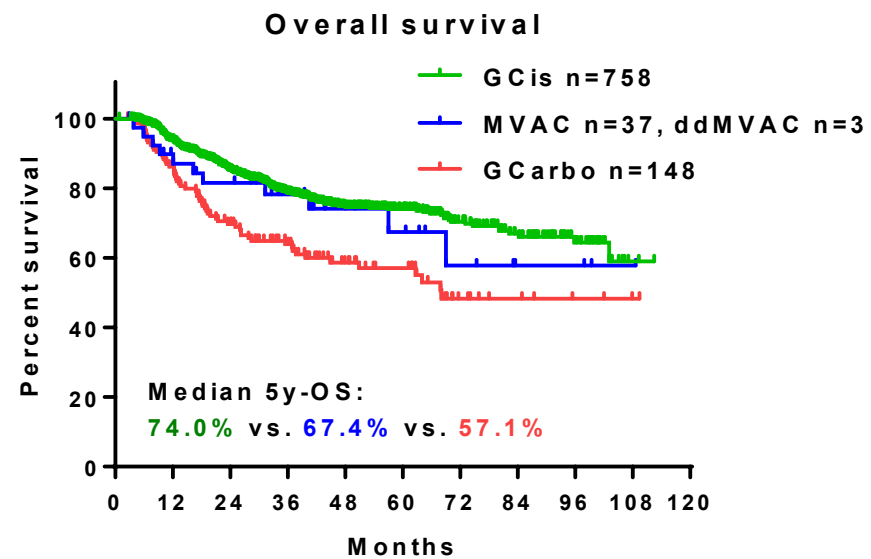

Fig.S4

A

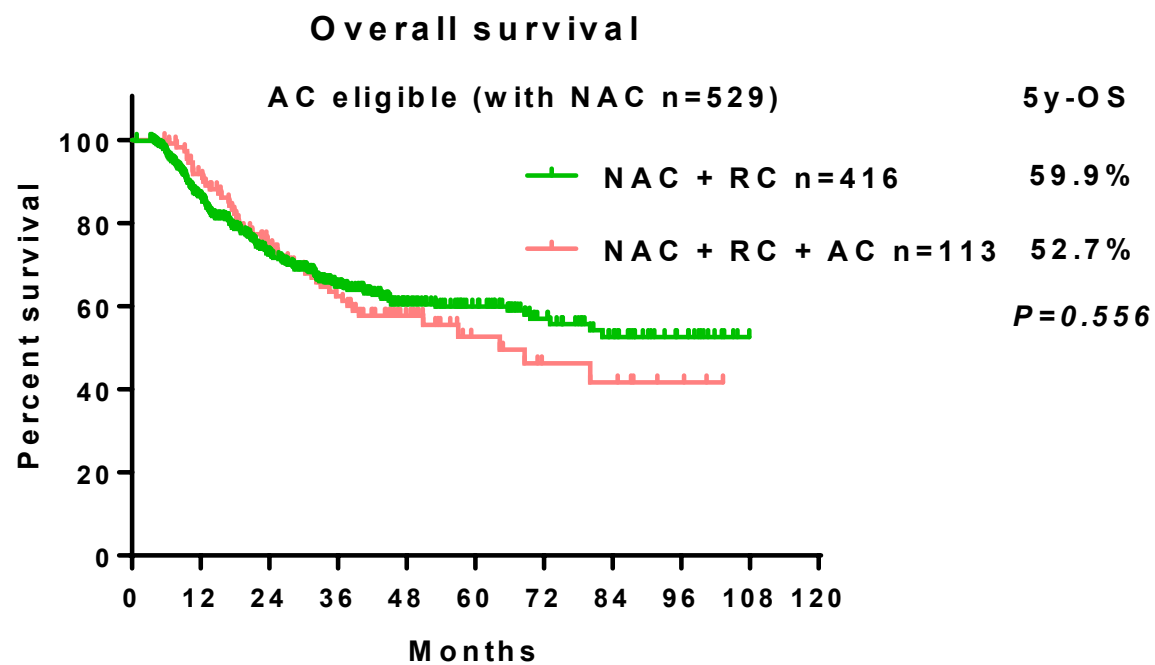

B

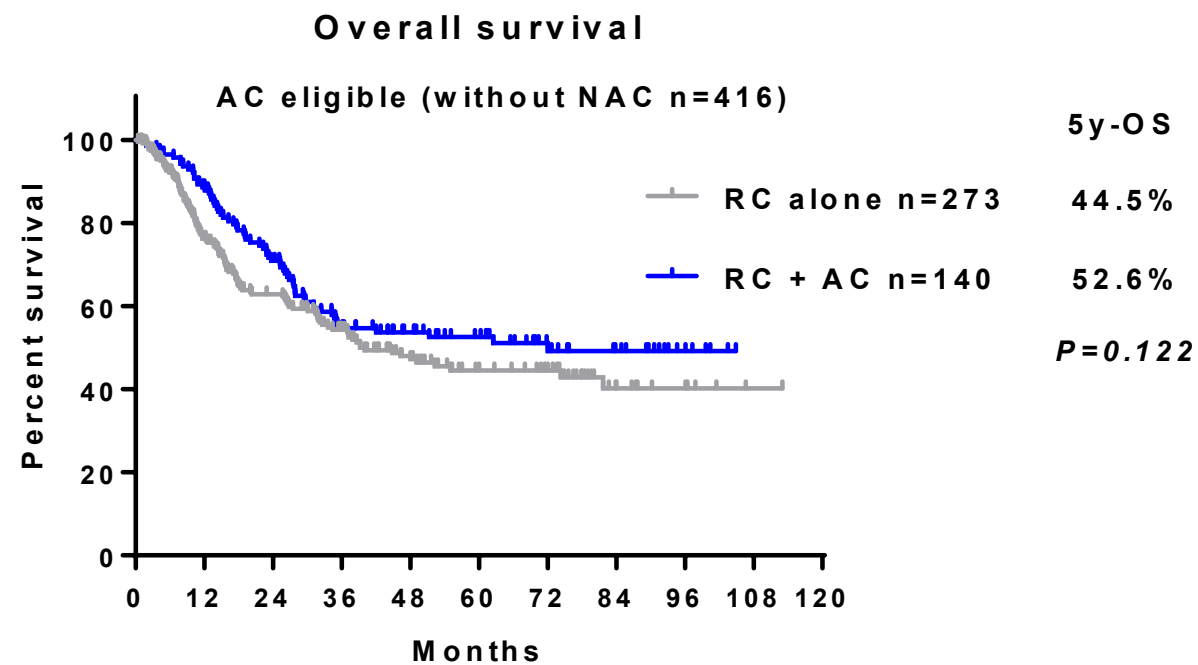

Fig. S5

A

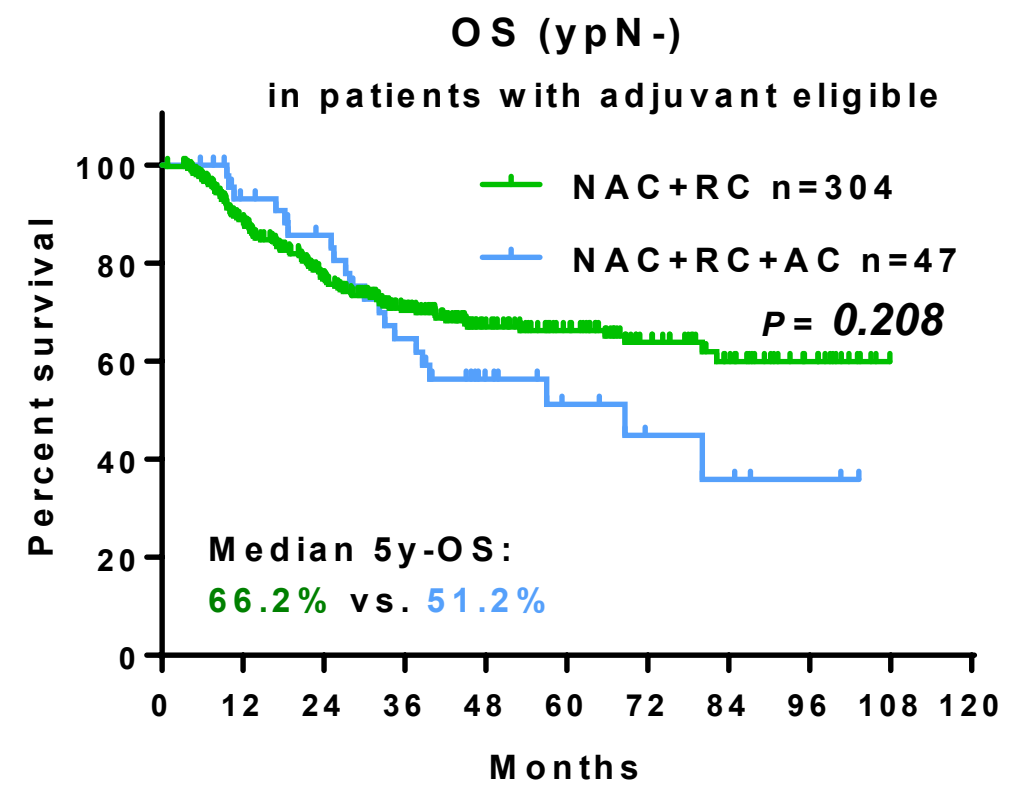

B

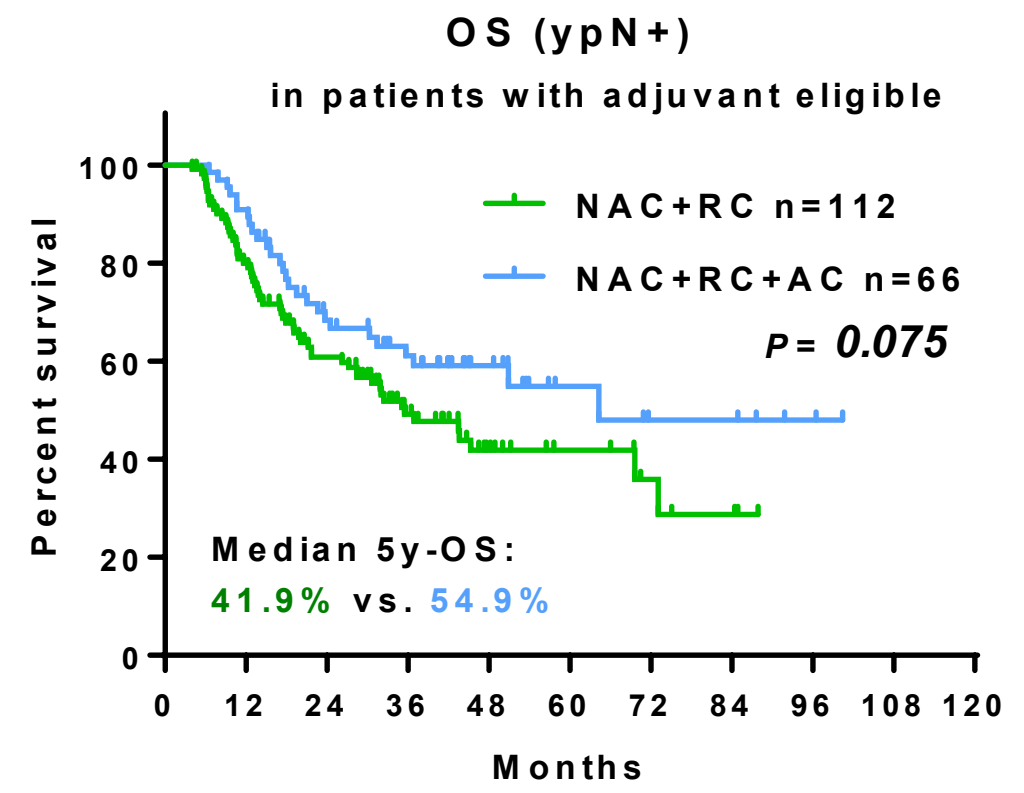

Supplement: Supplementary file 1 — Figures S1–S5. Figure S1. Overall survival (OS) analysis according to the number of neoadjuvant chemotherapy (NAC) cycles and regimens. (A) Overall survival (OS) stratified according to the number of NAC cycles: 2, 3, 4, and ≥ 5. (B) Comparison of the OS between the patients who received 2 and ≥ 3 cycles of cisplatin‐based NAC. (C) Comparison of the OS between the patients who received 2 and ≥ 3 cycles of carboplatin‐based NAC. Figure S2. Impact of delays in radical cystectomy (RC) on the OS of 939 patients who received neoadjuvant chemotherapy (NAC). (A) The median months from NAC to RC. We excluded seven cases without any data for time from NAC to RC. (B) Comparison of the overall survival between the patients with RC delays of ≤ 90 and > 90 days. Figure S3. Background difference of NAC regimens and its impact on OS. (A) Age difference between the GCis, MVAC/ddMVAC, and GCarbo. (B) eGFR difference between the GCis, MVAC/ddMVAC, and GCarbo. (C) OS difference between the GCis, MVAC/ddMVAC, and GCarbo. Figure S4. Subgroup analysis of the effect of adding adjuvant chemotherapy (AC) in patients eligible for AC. (A) Comparison of the OS between the patients who did and did not receive AC after NAC + RC. (B) Comparison of the overall survival between the patients who did and did not receive AC after RC. Figure S5. Comparison of OS in patients with ypN− or ypN+ between the NAC + RC and NAC + RC + AC who were eligible for AC. (A) Comparison of OS in patients with ypN− between the NAC + RC and NAC + RC + AC. (B) Comparison of OS in patients with ypN+ between the NAC + RC and NAC + RC + AC. [file CAM4-14-e70782-s001.pdf]
